# Endoscopic optical coherence tomography with wide field-of-view for the morphological and functional assessment of the human tympanic membrane

**DOI:** 10.1117/1.JBO.24.3.031017

**Published:** 2018-12-04

**Authors:** Lars Kirsten, Martin Schindler, Joseph Morgenstern, Mikael Timo Erkkilä, Jonas Golde, Julia Walther, Pascal Rottmann, Max Kemper, Matthias Bornitz, Marcus Neudert, Thomas Zahnert, Edmund Koch

**Affiliations:** a Technische Universität Dresden, Carl Gustav Carus Faculty of Medicine, Anesthesiology and Critical Care Medicine, Clinical Sensoring and Monitoring, Dresden, Germany; b Technische Universität Dresden, Carl Gustav Carus Faculty of Medicine, Otorhinolaryngology, Dresden, Germany; c Technische Universität Dresden, Carl Gustav Carus Faculty of Medicine, Medical Physics and Biomedical Engineering, Dresden, Germany

**Keywords:** optical coherence tomography, endoscopic imaging, otolaryngology

## Abstract

An endoscopic optical coherence tomography (OCT) system with a wide field-of-view of 8 mm is presented, which combines the image capability of endoscopic imaging at the middle ear with the advantages of functional OCT imaging, allowing a morphological and functional assessment of the human tympanic membrane. The endoscopic tube has a diameter of 3.5 mm and contains gradient-index optics for simultaneous forward-viewing OCT and video endoscopy. The endoscope allows the three-dimensional visualization of nearly the entire tympanic membrane. In addition, the oscillation of the tympanic membrane is measured spatially resolved and in the frequency range between 500 Hz and 5 kHz with 125 Hz resolution, which is realized by phase-resolved Doppler OCT imaging during acoustical excitation with chirp signals. The applicability of the OCT system is demonstrated *in vivo*. Due to the fast image acquisition, structural and functional measurements are only slightly affected by motion artifacts.

## 1 Introduction

The middle ear consists of the tympanic membrane and the ossicles, and ensures the transduction of sound from the auditory canal to the inner ear. Thus, diseases of the middle ear are often accompanied by hearing loss, especially in case of Eustachian tube dysfunction, acute or chronic otitis media, or otosclerosis. In the clinical routine, the diagnosis of pathological alterations in the middle ear is carried out using otoscopy or ear microscopy, audiometry, and tympanometry in order to assess morphological and functional changes of the middle ear.

An emerging technique for the investigation of the middle ear is optical coherence tomography (OCT),^1^ which is already established in other biomedical disciplines, such as ophthalmology, for noninvasive imaging of near-surface tissue. OCT is an interferometric measurement technique, utilizing broadband near-infrared light for cross-sectional imaging with a high spatial resolution in the range of several micrometers. Thus, OCT can be beneficially utilized for visualizing the middle ear morphology and it allows the spatially resolved measurement of the tympanic membrane thickness.^2^^,^^3^ Other studies have demonstrated that OCT can be used for imaging cholesteatoma^4^ and tympanosclerosis.^5^ In middle ear surgery, OCT has been utilized for the assessment of ossicles and middle ear prostheses.^6^ A further prominent application is the detection and classification of bacterial biofilms and middle ear fluids^7^^–^^11^ behind the tympanic membrane, which is important for the diagnosis and treatment of otitis media. There, OCT seems to be able to differentiate between serous and mucous middle ear effusions, being relevant for choosing appropriate therapeutic strategies. This has been addressed by Monroy et al.,^10^ who presented an approach for the determination of the viscosity of middle ear effusions by measuring diffusion coefficients.

In addition, phase-resolved Doppler OCT can be used for the investigation of the middle ear function by means of measuring the oscillation of the tympanic membrane and the ossicles, respectively, during acoustical excitation. This has been demonstrated in animal models^12^^,^^13^ and human middle ears.^14^^–^^16^ Most functional measurements are carried out with pure tone stimuli^12^^–^^15^ or multisinus,^17^ typically in the range between 500 Hz and 6 kHz, which covers the frequency range for speech perception being most relevant in diagnostics. In a previous *ex vivo* study,^16^ we demonstrated on human temporal bone with removed auditory canal that the oscillation of the tympanic membrane can be measured with high frequency resolution and spatially resolved within a short acquisition time of a few seconds when using an acoustical chirp excitation. Doppler OCT can visualize typical oscillation patterns of normal human tympanic membranes and it can detect characteristic changes in the oscillation behavior during pathological alterations. In case of a simulated Eustachian catarrh, it is observed that characteristic oscillation modes are shifted to higher frequencies due to the depression in the tympanic cavity.^18^ Furthermore, Doppler OCT could improve the detection of fluids in the tympanic cavity, since the presence of transparent nonscattering fluids can be indirectly confirmed due to the reduced oscillation amplitude of the tympanic membrane.^9^ In comparison to other functional measurement techniques, as holography^19^ or scanning laser Doppler vibrometry,^20^ OCT combines the advantages of three-dimensional, high-resolution imaging, and functional diagnostics, which are both features supporting established diagnostic tools, as otoscopy, tympanometry, and audiometry.

The *in vivo* application of OCT at the middle ear is challenging due to the curved auditory canal, which complicates the optical access to the tympanic membrane. One approach is to perform OCT through ear microscopes^21^ or ear specula^5^^,^^8^^,^^11^^,^^22^ with a long working distance being comparable with the length of the ear canal of ∼3  cm. In this case, the field-of-view for OCT is limited to a few millimeters and is not sufficient for scanning the entire tympanic membrane. MacDougall et al.^17^ and Park et al.^23^ presented optical solutions reaching an extended field-of-view up to 7 mm.^23^ An alternative approach for examining the middle ear is endoscopic OCT,^13^^,^^24^ realized with gradient-index (GRIN) optics providing a field-of-view up to 4 mm for imaging middle ear structures in mice^13^ or up to 10 mm for imaging human tympanic membranes.^24^

Endoscopic OCT can facilitate *in vivo* imaging, because the curved auditory canal is less impairing and the wide field-of-view allows the assessment of nearly the entire tympanic membrane. In particular, the functional assessment of the tympanic membrane could benefit from the opportunity of endoscopic imaging to visualize most parts of the tympanic membrane. In order to address this issue, we present functional endoscopic OCT imaging of the human tympanic membrane *in vivo* with a field-of-view of 8 mm within this proof of concept study. The specialized measurement protocol with acoustic chirp excitation allows a short acquisition time of 6.4 s for measuring the oscillation of the tympanic membrane between 500 Hz and 5 kHz, emphasizing the applicability under *in vivo* conditions. Additionally, it is demonstrated *ex vivo* on a human temporal bone specimen that OCT imaging is mostly unaffected by motion artifacts.

## 2 Materials and Methods

### 2.1 Endoscopic Optical Coherence Tomography System

**Fig. 1:**
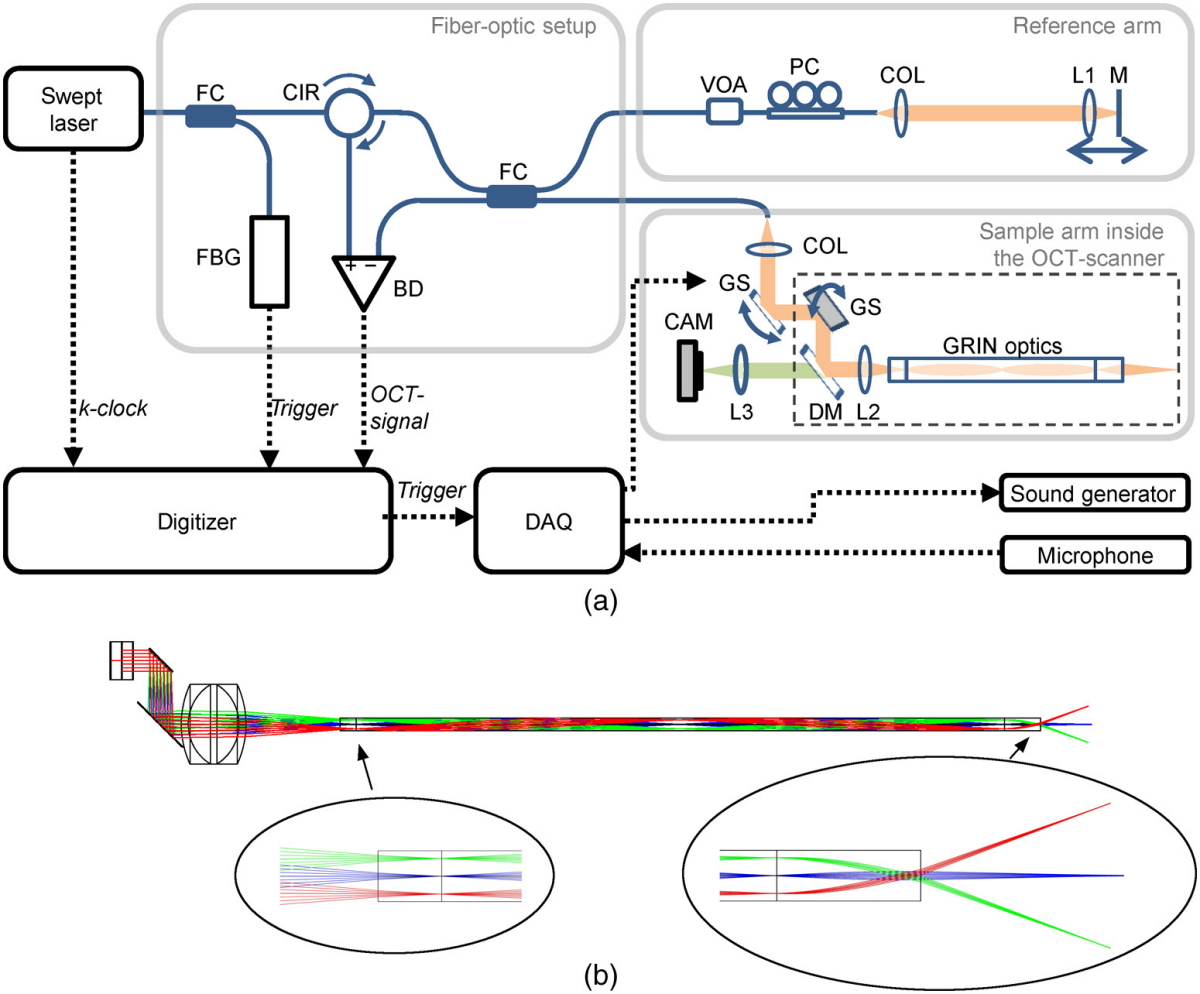
(a) OCT setup consisting of: BD, balanced detector; CAM, VIS-camera; CIR, circulator; COL, fiber collimator; DAQ, multifunction I/O device; DM, dichroic mirror; FBG, fiber Bragg grating trigger module; FC, fiber coupler; GRIN, gradient-index; GS, galvanometer scanner (driver not shown); L1–L3, lenses; M, mirror; PC, polarization controller; and VOA, variable optical attenuator. (b) Optic simulation of the endoscope optics, corresponding to the dashed box in (a).

The swept source OCT system, which is depicted in Fig. 1, utilizes a tunable laser (Axsun Technologies) with a repetition rate of 50 kHz and a center wavelength of 1300 nm. The spectral sweep range is up to 120 nm, leading to an axial resolution of 14  μm in air. The interferometer is implemented using a fiber-optic coupler (TW1300R5A2, Thorlabs) with 50% coupling ratio and separated optical setups for the sample arm (partly incorporated into the endoscopic OCT-scanner) and the reference arm, respectively. The fiber components consist of SMF-28e single-mode fiber.

The reference arm contains a variable optical attenuator (VOA50-APC, Thorlabs) for adjusting the optical power and a polarization controller (PLC-900, Thorlabs). Light, which is reflected at the mirror in the reference arm, propagates back to the fiber-optic coupler. In the endoscopic OCT scanner, the collimated laser beam (fiber-optic collimator 60FC-M15-37, Schäfter + Kirchhoff) is deflected by two galvanometer scanners and one dichroic mirror, before being focused with lens L2 [20-mm focal length; Hastings Triplet, 45-251, Edmund Optics) on a GRIN optics (GRINTECH GmbH) with 2-mm diameter, which is composed of a 2.5-mm glass spacer, a 1-pitch GRIN0 relay lens (GT-IFRL-200-100-10-NC), and a GRIN objective lens (GT-IFRL-200-010-50-C5(1)], as depicted in Fig. 1(b). After passing the GRIN optics, the laser beam is focused on the sample with a numerical aperture of 0.015. The GRIN optics has a transmission of 40% for both directions together, meaning back and forth.

The working distance (focus position) was adjusted to be 8 mm behind the last lens and the field-of-view is 8 mm as well. For optimized imaging of the tympanic membrane, the reference arm length was adjusted so that the focus position is located in the OCT images in a depth of 1.5 mm. The depth of focus (double Rayleigh length) amounts to 3.7 mm. Thus, it is possible to visualize most parts of the tympanic membrane within the OCT image depth range of 5 mm, as presented below (Fig. 4). The lateral resolution was measured with a 1951 USAF target to be 40  μm, giving the smallest lateral distance, which can be resolved.

The glass spacer is necessary, because in the case without glass spacer the air–glass interface of the GRIN relay lens would be in the focus of the lens L2 leading to a dramatically decreased signal-to-noise ratio due to the Fresnel-reflection at the air–glass surface. The glass spacer has improved the sensitivity by 23 dB. The sensitivity of the OCT system was measured to be −99  dB for a power of 1.4 mW on the sample. This sensitivity value was reached with adjusting the polarization controller in the reference arm. Sensitivity means the sample reflection intensity, compared to an ideal mirror, which yields a signal-to-noise ratio of 1 in the A-scan.^25^

The light, which is backscattered in the sample, is propagating back to the fiber-optic coupler and is interfering with the reference light. The OCT interference spectrum is acquired using a balanced detector and a high-speed digitizer (ATS9360, Alazar Technologies). The sample clock of the laser and a separate wavelength trigger (utilizing a fiber Bragg grating) is used for k-linear detection and A-scan triggering, respectively. Depth profiles of reflectivity, referred to as A-scans, are calculated out of the interference spectra mainly by Fourier transform. Cross-sectional images (B-scans) and volume scans are acquired during beam deflection using the galvanometer scanners, which causes a fan-shaped scan pattern at the distal end of the endoscope, as shown in Fig. 1(b). There, exemplary beam paths are shown, which do not correspond to the maximum beam deflection. For larger beam deflection, the beam would not enter completely the spacer at the GRIN optics. This is in agreement with the observed shadowing at the edge of the field-of-view.

An additional multifunction I/O-device provides the output voltages for the galvanometer scanner drivers and is used for synchronizing the acoustical measurements in case of functional OCT-imaging. The dichroic mirror in the endoscope allows the simultaneous acquisition of video endoscopic images. The lateral resolution for video endoscopy was measured to be 32  μm.

**Fig. 2:**
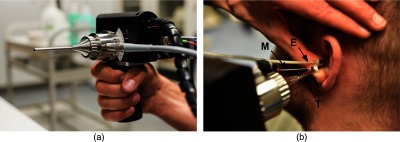
(a) Endoscopic OCT scanner with a 3.5-mm endoscopic tube diameter for the insertion into the auditory canal. (b) Application of the OCT scanner during an examination. For functional measurements, the flexible tube of an ear phone (E) and the flexible tube of a probe microphone (M) were additionally inserted after they were attached to the endoscopic tube (T). The auditory canal was sealed with a foam plug.

The OCT probe [Fig. 2(a)] is a self-built endoscopic OCT-scanner, whose endoscopic tube has an outer diameter of 3.5 mm. The GRIN optics with a protective metal tube is arranged in the middle of the endoscopic tube and is surrounded by optical fibers, which are connected to a cold-light source and ensure the illumination of the area imaged. For measuring the tympanic membrane oscillation, an ear phone (Ear-tone 3A, E-A-R Auditory systems) and a probe microphone (ER-7C, Etymotic Research) are attached to the OCT-probe. In order to measure the sound pressure next to the tympanic membrane, the probe microphone was positioned at the distal end of the endoscopic tube. The ear canal was sealed with a foam plug.

### 2.2 Measurement Principle

For the three-dimensional visualization of the tympanic membrane, a volume scan consisting of 640×640 A-scans was acquired within 8.2 s. A depth projection was used for obtaining overview OCT images with similar view on the tympanic membrane as in video endoscopy. Due to the fan-shaped scan pattern, a distortion of the sample would occur in the cross-sectional OCT images, if A-scans would be arranged in parallel. The B-scans presented in this paper have, therefore, been composed using a coordinate transformation from spherical coordinates to Cartesian coordinates, where the pixel size in the final B-scans was set to 8  μm in all dimensions (in air).

An additional scan pattern was used for the measurement of the tympanic membrane oscillation. The measurement was carried out on a grid of 25×25 points, which covers the same area as imaged by the volume scans. At each grid point, an M-scan consisting of 512 A-scans was acquired without moving the galvanometer scanners. Simultaneously, the tympanic membrane was acoustically excited using chirp stimuli, which covered the frequency range between 500 Hz and 5 kHz. The sound pressure level measured in the ear canal was 95.8 dBSPL. The acquisition time for one M-scan amounts to 10.24 ms and the total acquisition time for the entire 25×25 grid is accordingly 6.4 s.

**Fig. 3:**
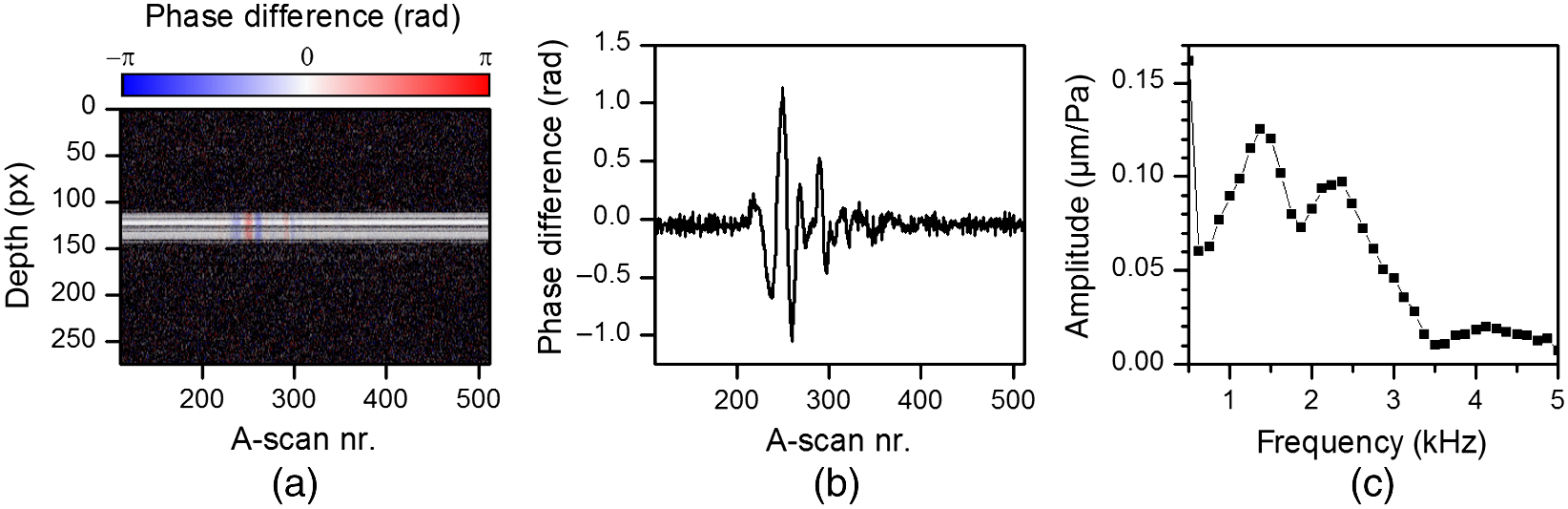
(a) Exemplary M-scan with color-coded phase difference, (b) phase difference $\Delta \varphi_{z}$ extracted from the depth with maximum intensity and averaged over 5 pixels in depth direction, and (c) the calculated oscillation amplitude being normalized to the sound pressure.

Using phase-resolved Doppler evaluation,^26^ the phase difference between adjacent A-scans was calculated, which is proportional to the velocity of the tympanic membrane. A part of an exemplary M-scan is depicted in Fig. 3(a), where the phase difference Δϕ is color-coded. Since the positioning of the galvanometer scanners at the beginning of the M-scans produces visible oscillations in the M-scan and additional phase signals hampering the evaluation, the first 112 A-scans of each M-scan were discarded. Thus, the M-scan, shown in Fig. 3(a), consists of the 400 A-scans left. Since the chirp is repeated every 400 A-scans, it is ensured that the part of the M-scan used for further analysis contains a complete period of acoustical excitation.

The time course of the phase difference $\Delta \varphi_{z}$ [Fig. 3(b)] was extracted from the depth having the highest intensity value (using a threshold to suppress noise) and was averaged over 5 pixels in depth. Afterward, a Fourier transform was applied and the axial velocity amplitude $v_{\text{max}}$ can be calculated according to Eq. (1) using the relationship between phase difference and axial velocity component described by Walther et al.,^26^ where λ is the center wavelength, $f_{A}$ is the A-scan rate, $f_{\text{osc}}$ is the oscillation frequency, and n is the refractive index (being n=1 for the case of air)

$$v_{\max}(f_{\text{osc}})=\frac{\lambda \cdot f_{A}}{4\pi \cdot n}\cdot \mathrm{FFT}(\Delta \varphi_{z}).$$ (1)

The oscillation amplitude $A(f_{\text{osc}})$ can be calculated as follows:

$$A(f_{\text{osc}})=\frac{1}{2\pi \cdot f_{\text{osc}}}\cdot v_{\max}(f_{\text{osc}}).$$ (2)

**Fig. 4:**
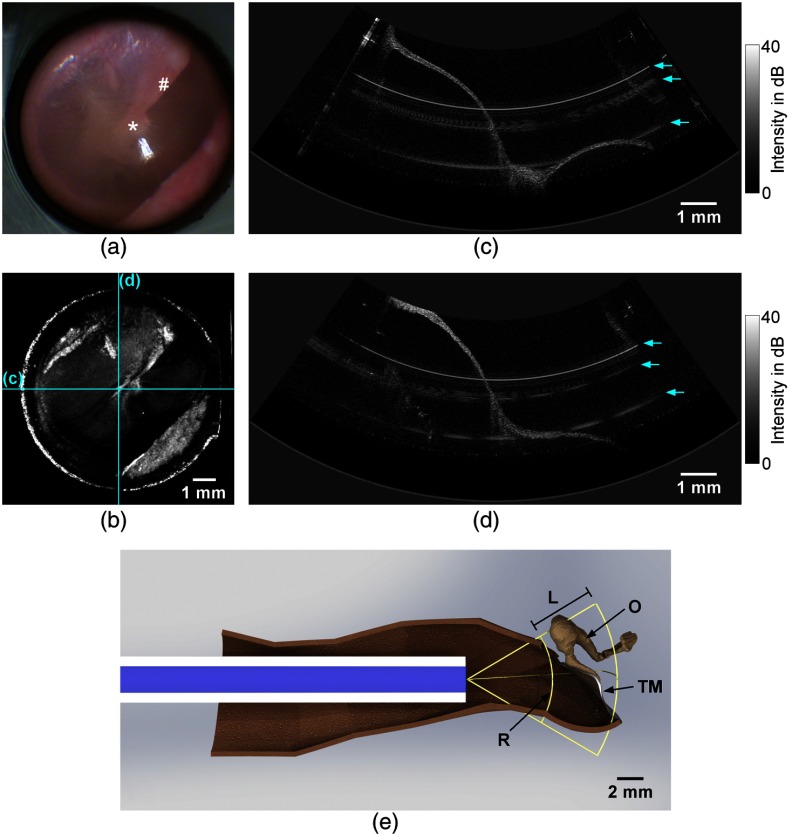
The imaging results show the tympanic membrane of a right ear *in vivo*: (a) video endoscopic image showing the pars tensa with the umbo (*) and the manubrium of malleus (#) and (b) corresponding depth projection of an OCT volume scan. The scale bar in (b) is applicable for the working distance of 8 mm. (c) and (d) Horizontal and vertical central OCT cross sections, corresponding to the fast and slow scan directions. The locations of the cross sections are indicated in the depth projection (b). The arrows in (c) and (d) mark the positions of circular artifacts. (e) Computer-aided design (CAD) model showing the measurement situation with the endoscope being positioned inside an auditory canal: (TM) tympanic membrane, (O) ossicles, (R) reference plane corresponding to the upper border of the OCT image in (d), and (L) OCT image depth range of 5 mm. The OCT image area is spanned by the reference plane R and the image depth range L.

The measured phase difference gives the axial component of the velocity or oscillation amplitude, respectively, meaning in the direction of the incident laser beam. In general, the determined phase difference depends on the observation angle, which is the angle between the laser beam and the oscillation direction of the tympanic membrane. The consideration of the observation angle would require precise prior knowledge of the spatially resolved oscillation direction of the tympanic membrane. Using the OCT cross sections (Fig. 4), the observation angle can only be estimated to be typically in the range between 0 deg and 60 deg, depending on the position on the tympanic membrane and assuming an oscillation perpendicular to the surface. Consequently, the measured oscillation amplitude in Eq. (2) could underestimate the actual oscillation amplitude by a factor of 2 for the highest expected observation angle (60 deg). This effect is expected to be in the range of interindividual variations and is expected to be small compared to clinically relevant alterations of the oscillation amplitude. Hence, the influence of the observation angle was neglected in the calculation of the oscillation amplitude.

The frequency response of the sound pressure $p(f_{\text{osc}})$ applied was calculated out of the microphone signal by Fourier analysis as well

$$p(f_{\text{osc}})=\mathrm{FFT}(p).$$ (3)

Finally, the transfer function [Fig. 3(c)], which describes the oscillation amplitude being normalized to the sound pressure, was calculated as the ratio of both frequency responses (T=A/p). The length of 400 A-scans per M-scan results in a frequency increment of 125 Hz after Fourier transform. At low frequencies, the sealing of the auditory canal is imperfect, which yields to an underestimated measured sound pressure and, therefore, causing an overestimated normalized oscillation amplitude, as visible in Fig. 3(c) at 500 Hz.

This algorithm provides the transfer functions for all grid points, which allowed the reconstruction of the tympanic membrane oscillation for frequencies within the range of excitation, from 500 Hz to 5 kHz, with 125-Hz frequency increment. In order to calculate the tympanic membrane oscillation, a periodical oscillation with the amplitudes and phases at the corresponding frequency was assumed. Before displaying the oscillation maps or videos, grid points showing no information or noise were set to zero amplitude using a semiautomatic mask.

### 2.3 Imaging Procedure

Structural and functional endoscopic OCT is demonstrated *in vivo* at the right ear of a volunteer (28 years, male), where pathological alterations at the middle ear had been excluded by an ENT specialist. The subject was in sitting position while the examiner stood beside him. The endoscope was heated by a mirror warmer to prevent misting up and was inserted with control via VIS camera image and OCT preview. After positioning, the ear canal was sealed by a foam plug and image acquisition was started using a foot switch. The morphological measurement and the functional measurement were carried out consecutively. Each image acquisition was started separately using a foot switch. During the examination and also during the data acquisition, the examiner controlled the positioning of the endoscope by the white light camera image. Additionally, the probe position was controlled by the cross-sectional preview OCT images between the separate parts of the measurement. The examination was finished within 1 min, where the acquisition of a volume scan and a functional measurement took 8.2 and 6.4 s, respectively.

*Ex vivo* imaging was conducted on a fresh right cadaveric temporal bone (female, 67 years) that was preserved in 4°C saline solution after extraction. Pathological changes of the middle ear were excluded using light microscopy. Images were acquired within 13 days after death. For comparing fixed and hand-held measurement, the specimen was fixed in a position comparable to a sitting patient. The endoscope was mounted on a translation stage via an articulated arm. The endoscope was then inserted into the outer ear canal and manually adjusted before final positioning was achieved using the translation stage. After acquiring images in the fixed setting, the measurement was repeated in hand-held setting at the same position.

## 3 Results and Discussion

The results of video endoscopy and structural OCT imaging are shown in Fig. 4. The field-of-view of 8 mm at a working distance of 8 mm allowed the visualization of nearly the entire tympanic membrane. The funnel-shaped tympanic membrane is connected to the manubrium of malleus between the umbo, which is the deepest point of the funnel, and the lateral process. The manubrium of malleus is visible in the endoscope image behind the translucent tympanic membrane [Fig. 4(a)]. In comparison, Fig. 4(b) shows the depth projection (mainly average intensity) of the volume scan acquired, which shows an overview OCT image of the field-of-view visualizing the pars tensa and the manubrium of malleus as well. Imaging further parts of the tympanic membrane, as the pars flaccida, would require a repositioning of the endoscope. The anterior and lower wall of the auditory canal limits slightly the view on the antero-inferior quadrant. The visualization of the ossicles, especially the stapes, behind the tympanic membrane could be possible in future by increasing the imaging depth, which depends on several parameters, mainly on the coherence length of the laser and the sampling rate.

The cross-sectional OCT images [Figs. 4(c) and 4(d)] were extracted out of the volume scan and represent the horizontal [Fig. 4(c)] and vertical [Fig. 4(d)] directions, which correspond to the fast and slow scan directions, respectively. The funnel-like shape of the tympanic membrane is visible in both cross sections, because they are intersecting each other approximately at the umbo. Assuming a refractive index for the tympanic membrane tissue of ∼1.44,^2^ the tympanic membrane thickness can be estimated. The tympanic membrane examined has a minimum thickness of about 70  μm and the thickness increases toward the annulus and at the manubrium of malleus. This is in good agreement with previous investigations,^2^ where the thickness of the tympanic membrane is measured spatially resolved with OCT. There, the thickness varies between 60 and 90  μm at the pars tensa and becomes higher than 100  μm toward the annulus and at the manubrium of malleus.

The OCT image range of 5 mm in depth was sufficient for imaging nearly the entire tympanic membrane. Since the tympanic membrane is oriented in the auditory canal approximately at a 40-deg angle with respect to the sagittal plane, postero-superior parts of the tympanic membrane, which were located between the zero-path position (upper border of the OCT image) and the endoscope, are visualized as mirrored signals in the OCT image, although they are located out of the image range. This effect is visible in the OCT cross sections and in the OCT depth projection as well. An increased image range could facilitate the OCT imaging in future.

The upper border of the OCT image corresponds to the reference plane, which is the position of zero path difference in the interferometer. The distance between the endoscope and this reference plane is 6.5 mm. The small parts of the tympanic membrane, which are visible as mirror signals, have a smaller distance to the endoscope, which is typically at least 5 mm. This safety distances ensure that the endoscope is not touching the tympanic membrane during the examination.

In order to illustrate the position of the endoscope in the auditory canal, Fig. 4(e) shows a CAD model of the measurement situation. There, the geometry of the auditory canal was assumed as determined by Stinson and Lawton.^27^ The sectional view in Fig. 4(e) shows the expected position and orientation of the tympanic membrane in the OCT image area, which is in agreement with the cross section in Fig. 4(d). The CAD model confirms that nearly the entire tympanic membrane can be visualized and that the safety distance is typically at least 5 mm.

Some circular artifacts are visible in the cross sections, which are caused by reflexes at surfaces or interfaces of the optical components, and are therefore, visible at certain depth positions. A coherence revival effect,^28^ caused by the laser operation principle, allows the interference between interfaces, whose distance is larger than the image depth and larger than the actual coherence length of the laser. Due to the several optical components in the interferometer, it is difficult to avoid these artifacts completely.

**Fig. 5:**
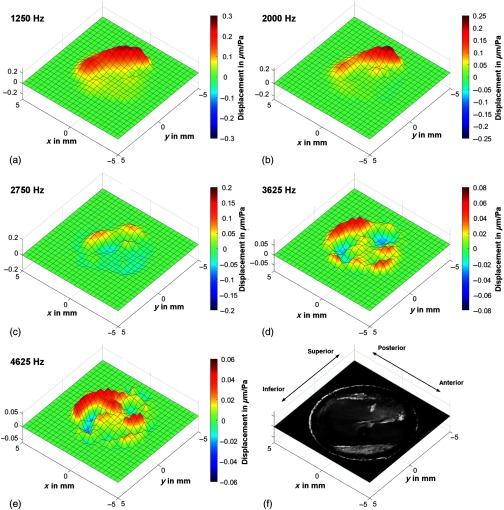
(a)–(e) Exemplary oscillation patterns *in vivo* at characteristic frequencies, which were calculated in postprocessing out of one functional OCT measurement, show the deflection of the tympanic membrane. The displacement scale and color map are adjusted to the maximum amplitude of each oscillation pattern. (f) Depth projection of the corresponding OCT volume scan for comparison. The x and y scales are applicable for the working distance of 8 mm.

The functional examination by means of Doppler OCT revealed the oscillation behavior of the tympanic membrane *in vivo* for frequencies within the range of acoustical excitation, from 500 Hz to 5 kHz. Figure 5 shows the oscillation for representative frequencies, which correspond to typical oscillation modes of the tympanic membrane. In addition, Video 1 (Fig. 7) is provided in Sec. 5, which shows the oscillation for the frequencies presented in Fig. 5.

In Fig. 5, the oscillation maps show time points, which give an impression of the oscillation characteristics. For most frequencies in Fig. 5, the selected time points coincide with the time of maximum elongation. Only in Fig. 5(c), the oscillation map does not show the maximum elongation, instead the time point, where the out-of-phase oscillation is more clearly visible. For plotting the oscillation maps, a linear interpolation was used between neighboring grid points and the elongation displayed was scaled up in order to facilitate the visualization. Accordingly, different scales have been used for the oscillation maps displayed in Fig. 5. A semiautomatic mask was applied, which sets the amplitude to zero for points being outside the circular field-of-view or showing a noise-like transfer function. In Fig. 5(f), the depth projection of the OCT volume scan is displayed for comparison. There, the manubrium of malleus is visible as a bright line from the umbo toward anterior–superior direction.

Figure 5(a) shows the oscillation at 1250 Hz, which is approximately the resonance frequency, where the tympanic membrane is oscillating in phase and exhibits the highest amplitude. As expected, the oscillation amplitude is reduced at the part of the pars tensa, which is connected to the manubrium of malleus. The oscillation pattern is similar for frequencies below 1250 Hz, but exhibiting smaller oscillation amplitude. The oscillation for the entire frequency range from 500 Hz to 5 kHz is provided in Sec. 5 (Fig. 8, Video 2).

At a frequency of 2000 Hz [Fig. 5(b)], the oscillation amplitude has its maximum in the posterior part as well but a travelling wave toward the superior part of the pars tensa and toward the pars flaccida is visible. At higher frequencies, the oscillation is more complex, e.g., showing an out-of-phase oscillation mainly between the posterior and anterior part at 2750 Hz. Increasing the frequency further [Figs. 5(d)–5(e)], the oscillation is becoming similar to circular oscillation modes. Multiple oscillation maxima are visible, which are mostly circularly arranged. The maximum amplitude is reduced from 0.28  μm/Pa at 1250 Hz to 0.06  μm/Pa at 4625 Hz. The tympanic membrane oscillation measured is in agreement with previously published results based on Doppler OCT^16^ or digital holography,^19^ which described similar oscillation amplitudes and oscillation patterns.

The spatial resolution for the functional OCT measurement depends on the grid spacing. At a working distance of 8 mm, the distance between adjacent grid points is about 400  μm. This is sufficient for displaying the details of the oscillation patterns at frequencies up to 5 kHz, as visible in Fig. 5(e). As already discussed above, the part of the tympanic membrane being located between the endoscope and the upper edge of the image range is displayed mirrored. Those image parts exhibit a 180-deg phase shift in the Doppler signal, which was corrected taking into account that the tympanic membrane is oscillating in phase at frequencies below 1 kHz. Comparing the phases of all grid points with the phase of a correctly imaged M-scan allowed the selection of points exhibiting a phase jump of 180 deg.

The hand-held application provides a good flexibility with regard to the premises and the examination conditions. Unfortunately, some movement of both the subject and the examiner cannot be avoided. As the acquisition time for a single B-scan in fast scan direction is just 13 ms, there are no visible artifacts for this condition. However, the total acquisition time amounts several seconds, in particular 8.2 s for a volume scan and 6.4 s for the functional measurement. During this time, a motion of the endoscope with respect to the position of the tympanic membrane could also cause a distortion of the volumetric images. During the acquisition, the examiner can control the position of the endoscope by video endoscopy and avoids any motion as well as possible. Our results show that there are only minor motion artifacts in the 3-D images, especially in slow scan direction, as visible in Fig. 4(d). There, the tympanic membrane surface appears not as smooth as in Fig. 4(c), which is probably caused by the tremor.

With longer acquisition time, motion artifacts related to the tremor would become more likely. Therefore, the structural and functional measurements are carried out separately. In the time in between, the examiner can take a short break and the examiner can reposition the endoscope if necessary by the help of video endoscopy and OCT preview.

During the functional OCT measurements, the acquisition time for a M-scan is only 10 ms. Furthermore, the oscillation frequency of the tympanic membrane is in the range between 500 Hz and 5 kHz, which is about two orders of magnitude higher than the mean tremor frequency^29^^,^^30^ being in the range between 7 and 9 Hz. Thus, it is expected that the functional measurements, in particular the Doppler-OCT measurement and data evaluation, remain mainly unaffected by the motion of the examiner and the patient. Accordingly, no motion artifacts are visible in Fig. 5.

Because of the acquisition time of 6.4 s for a functional measurement, a strong motion of the endoscope during this acquisition time could distort the oscillation pattern by changing the direction of view. However, the distance between neighboring grid points on the oscillation maps is ∼0.4  mm. Motion related displacements of the endoscope up to this extent are, therefore, not expected to affect the oscillation pattern.

**Fig. 6:**
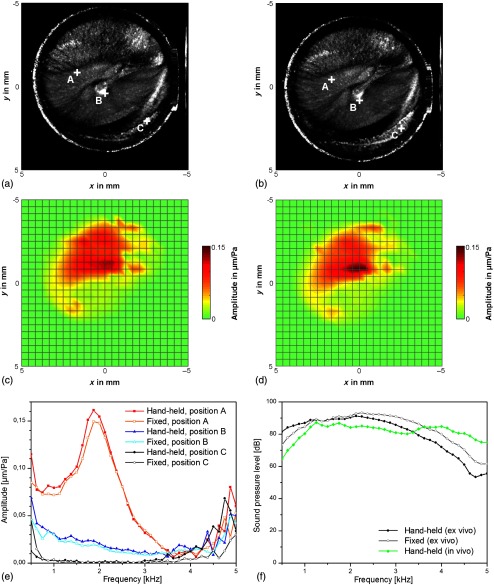
Comparison of *ex vivo* OCT measurements on a human temporal bone specimen with hand-held OCT-scanner (a), (c) and fixed OCT-scanner (b), (d), showing the depth projections of OCT volume scans (a), (b) and the oscillation amplitude at the frequency of 1.500 Hz (c), (d). The x and y scales are applicable for the working distance of 8 mm. (e) The normalized oscillation amplitude is shown for exemplary points A, B, and C marked in (a) and (b). The points A and B correspond to positions on the pars tensa. In order to demonstrate the detection limit, point C was selected to be outside the tympanic membrane, where no sound-induced oscillation is expected. (f) Sound pressure level for the *ex vivo* measurements with hand-held and fixed OCT-scanner and for the *in vivo* measurement presented in Fig. 5.

To investigate the influence of the motion on the functional measurements, it would be necessary to compare measurements with hand-held and fixed OCT scanner. Since a measurement with a fixed OCT scanner is not feasible *in vivo*, measurements with fixed and hand-held endoscope were carried out *ex vivo* on a human temporal bone specimen (Fig. 6), although this setting considers only for the motion of the examiner. Furthermore, it is expected that the extent of the motion artifacts and the quality of the measurement results can vary between different examiners and patients. These issues have to be addressed in a future patient study.

Figures 6(a) and 6(c) show the depth projection of a volume scan and the amplitude map for an exemplary frequency of 1.500 Hz for a measurement with hand-held OCT scanner. For comparison, Figs. 6(b) and 6(d) show the measurement results for the case with fixed OCT scanner. The measurements with fixed and hand-held endoscope revealed almost identical depth projections and oscillation patterns. This also demonstrates that the volumetric images can be acquired without visible distortions, which would have been related to the acquisition time of several seconds. A slight deviation in the orientation and position of the tympanic membrane is visible, which was caused by a different positioning of the endoscope.

In Fig. 6(e), the normalized oscillation amplitude is shown for exemplary grid points, whose positions are marked in the depth projections. The points A and B are located at the pars tensa, where only small deviations between hand-held and fixed configuration are visible in Fig. 6(e). In order to demonstrate the influence of the motion on the detection limit, point C was selected to be outside the tympanic membrane. There, no sound-induced oscillation is expected and the normalized amplitude should show the noise floor. For hand-held and fixed endoscope, the noise floor is similar between 0.7 and 3.4 kHz. This is in accordance with a previous study,^15^ where the noise floor was investigated for hand-held and fixed probe and no difference was measured in the frequency range above 1.5 kHz and only a slightly increased noise level could be measured below 1.5 kHz.

However, in Fig. 6(e), the noise floor for the normalized oscillation amplitude is increased for the case with fixed endoscope in the frequencies range below 0.7 kHz and the noise floor is increased for the case with hand-held endoscope for frequencies above 3.4 kHz. This is mainly caused by the different sound pressure levels [Fig. 6(f)], where the deviations between hand-held and fixed conditions are probably caused by a difference in the sealing of the auditory canal. Figure 6(f) shows also the sound pressure level for the *in vivo* measurement presented in Fig. 5, where the sound pressure level is significantly higher for the upper frequency range up to 5 kHz.

## 4 Conclusion

The endoscopic OCT system presented provides a wide field-of-view of 8 mm at a working distance of 8 mm, which allows the visualization of nearly the entire tympanic membrane. The spatial resolution is 16  μm in axial direction and 40  μm in lateral direction. Simultaneously, video endoscopic images can be acquired for the facilitated application in the auditory canal. For the assessment of the tympanic membrane function, a measurement protocol was utilized, which measures the tympanic membrane oscillation spatially resolved and resolved in frequency between 500 Hz and 5 kHz using phase-resolved Doppler OCT during acoustic excitation with chirp stimuli. Thus, the OCT system allows a morphological and functional examination of the human middle ear. The short acquisition times of 8.2 s for volume scans or 6.4 s for functional measurements facilitates the application *in vivo*. It was demonstrated that only minor motion artifacts are observed in the 3-D OCT image stack and that the Doppler OCT measurements are widely unaffected by motion artifacts. Thus, it is possible to determine the 3-D structure of the tympanic membrane and their oscillation patterns *in vivo*.

The wide-ranging imaging capability of OCT makes it a promising tool for diagnostic applications in otolaryngology, where OCT is not restricted to morphological imaging. Additionally, the investigation of the tympanic membrane oscillation is expected to facilitate the diagnosis of conductive hearing losses and sclerotic alterations like malleus head fixation. Furthermore, OCT allows the detection of pathological alterations behind the tympanic membrane, like cholesteatoma, the evaluation of middle ear effusion, and the coupling of prostheses after middle ear reconstruction.

## 5 Appendix

**Fig. 7:**
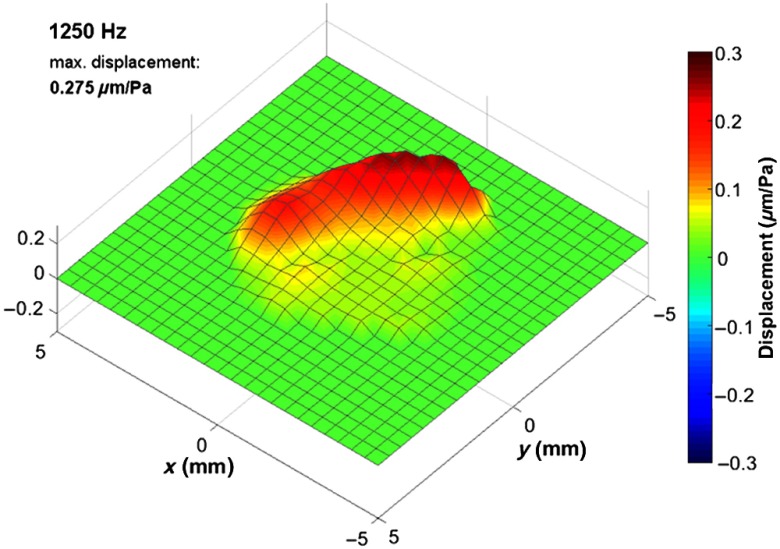
Oscillation of the tympanic membrane at exemplary characteristic frequencies, shown in Fig. 5, calculated in postprocessing out of one functional *in vivo* OCT measurement. (Video 1, MPEG, 7.4 MB [URL: https://doi.org/10.1117/1.JBO.24.3.031017.1]).

**Fig. 8:**
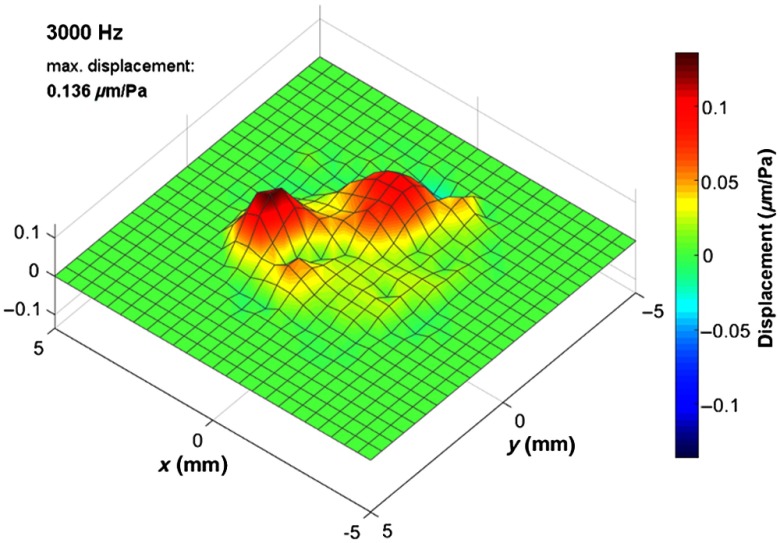
Oscillation of the tympanic membrane for frequencies within the range between 500 Hz and 5 kHz with 125-Hz resolution, calculated in postprocessing out of one functional *in vivo* OCT measurement. (Video 2, MPEG, 9.2 MB [URL: https://doi.org/10.1117/1.JBO.24.3.031017.2]).

In addition to Fig. 5, the oscillation of the tympanic membrane is shown in Fig. 7 for the characteristic frequencies, selected for Fig. 5, and in Fig. 8 for the frequency range between 500 Hz and 5 kHz.
